# Antimicrobial Resistance of *Escherichia coli* From Aquaculture Farms and Their Environment in Zhanjiang, China

**DOI:** 10.3389/fvets.2021.806653

**Published:** 2021-12-24

**Authors:** Cui-Yi Liao, Balamuralikrishnan Balasubramanian, Jin-Ju Peng, Song-Ruo Tao, Wen-Chao Liu, Yi Ma

**Affiliations:** ^1^College of Coastal Agricultural Sciences, Guangdong Ocean University, Zhanjiang, China; ^2^Department of Food Science and Biotechnology, College of Life Science, Sejong University, Seoul, South Korea

**Keywords:** antimicrobial resistance, aquaculture farm, *Escherichia coli*, multi-drug resistance, resistance genes

## Abstract

Antimicrobial resistance (AMR) has become a major concern worldwide. To evaluate the AMR of *Escherichia coli* in aquaculture farms of Zhanjiang, China, a total of 90 samples from the water, soil, and sediment of three aquaculture farms (farms I, II, and III) in Zhanjiang were collected, and 90 strains of *E. coli* were isolated for drug resistance analysis and AMR gene detection. The results indicated that the isolated 90 strains of *E. coli* have high resistance rates to penicillin, amoxicillin, ampicillin, tetracycline, compound sulfamethoxazole, sulfisoxazole, chloramphenicol, florfenicol, and rifampin (≥70%). Among these antimicrobial drugs, the resistance rate to rifampicin is as high as 100%. Among the isolated 90 strains of *E. coli*, all of them were resistant to more than two kinds of antimicrobial drugs, the number of strains resistant to nine kinds of drugs was the largest (19 strains), and the most resistant strain showed resistance to 16 kinds of antibacterial drugs. Regarding the AMR genes, among the three aquaculture farms, the most resistance genes were detected in farm II (28 species). The detection rate of *bla*_*TEM*_, *bla*_*CIT*_, *bla*_*NDM*_, *floR, OptrA, cmlA, aphA1, Sul2, oqxA*, and *qnrS* in 90 isolates of *E. coli* was high (≥50%). The detection rate of carbapenem-resistant genes, such as *bla*_*KPC*_, *bla*_*IMP*_, and *cfr*, was relatively lower ( ≤ 30%), and the detection rate of *mcr2* was the lowest (0). At least four AMR genes were detected for each strain, and 15 AMR genes were detected at most. Among them, the number of strains that carried 10 AMR genes was the largest (15 strains). Finally, a correlation analysis found that the AMR genes including *bla*_*TEM*_, *bla*_*CIT*_, *floR, OptrA, cmlA, aac(3)-II, Sul2, ereA, ermB, oqxB, qnrA, mcr1*, and *mcr2* had a high correlation rate with drug resistance (≥50%). To summarize, the 90 strains of *E. coli* isolated from water, surrounding soil, and sediment samples showed resistance to multi-antimicrobial drugs and carried various antimicrobial resistance genes. Thus, it is essential to strengthen the rational use of antimicrobial drugs, especially the amide alcohol drugs, and control the AMR in the aquaculture industry of Zhanjiang, China.

## Introduction

Currently, due to human demand for a variety of animal proteins, the aquaculture industry is developing rapidly (1). China has been one of the largest producers of aquatic products, and the coastal area is the main aquaculture base, including the coastal city of Zhanjiang (2). Because of poor resistance and susceptibility to disease of aquatic animals, antibiotics are widely used in aquaculture to prevent and treat bacterial diseases (1). As a result, the residual antibiotics in aquatic products pose a potential risk to food safety, and antibiotics excreted from aquatic animals are dispersed in the water and sediments, thus leading to the emergence of drug-resistant bacteria and antimicrobial resistance (AMR) genes in aquaculture farms and the surrounding environment (3, 4). Under the pressure of excessive use of antibiotics, the drug-resistant bacteria even appeared to demonstrate multi-drug resistance, which causes serious negative impacts on public health (5). Besides this, according to the report of Pruden et al. (6), the AMR genes are novel environmental pollutants. The existence of drug resistance genes is the key to the development of drug resistance in bacteria. The AMR genes not only spread vertically but also spread to other bacteria horizontally through genetic elements, increasing the number of drug-resistant strains and causing difficulties in the treatment of clinical diseases and infection in both humans and animals (7, 8).

*Escherichia coli* is a gram-negative and common conditional pathogenic bacteria in the intestines and environment of humans and animals (9). Pathogenic *Escherichia coli* results in intestinal diseases and infections of farm animals (10, 11). *Escherichia coli* also induces diseases in various aquatic animals, thereby causing serious economic losses for the aquaculture industry. In this context, the excessive use of antibacterial drugs in aquaculture has become inevitable, and aquaculture has become an important source of antibiotic-resistant strains and AMR genes of *E. coli* (1). However, the terrible thing is that the antibiotic-resistant bacteria can infect humans through the food chain or transfer AMR genes to human pathogens, leading to serious diseases such as meningitis, sepsis, and enterotoxemia in humans (12). Furthermore, the frequent application of similar antimicrobial drugs in the treatment of *E. coli* induced the diseases of animals (including aquatic animals) and humans nowadays, which makes it difficult to find an effective antimicrobial agent in case of bacterial infections in humans (13). However, little is known about the drug resistance and the distribution of AMR genes of *E. coli* isolates from aquaculture farms and their surroundings in Zhanjiang, China. Therefore, to provide basic data for a better understanding of AMR in aquaculture, the present study was conducted to evaluate the distribution of AMR genes and analyze the drug resistance of *E. coli* in aquaculture farms of Zhanjiang, China.

## Materials and Methods

### Sampling

**Figure 1:**
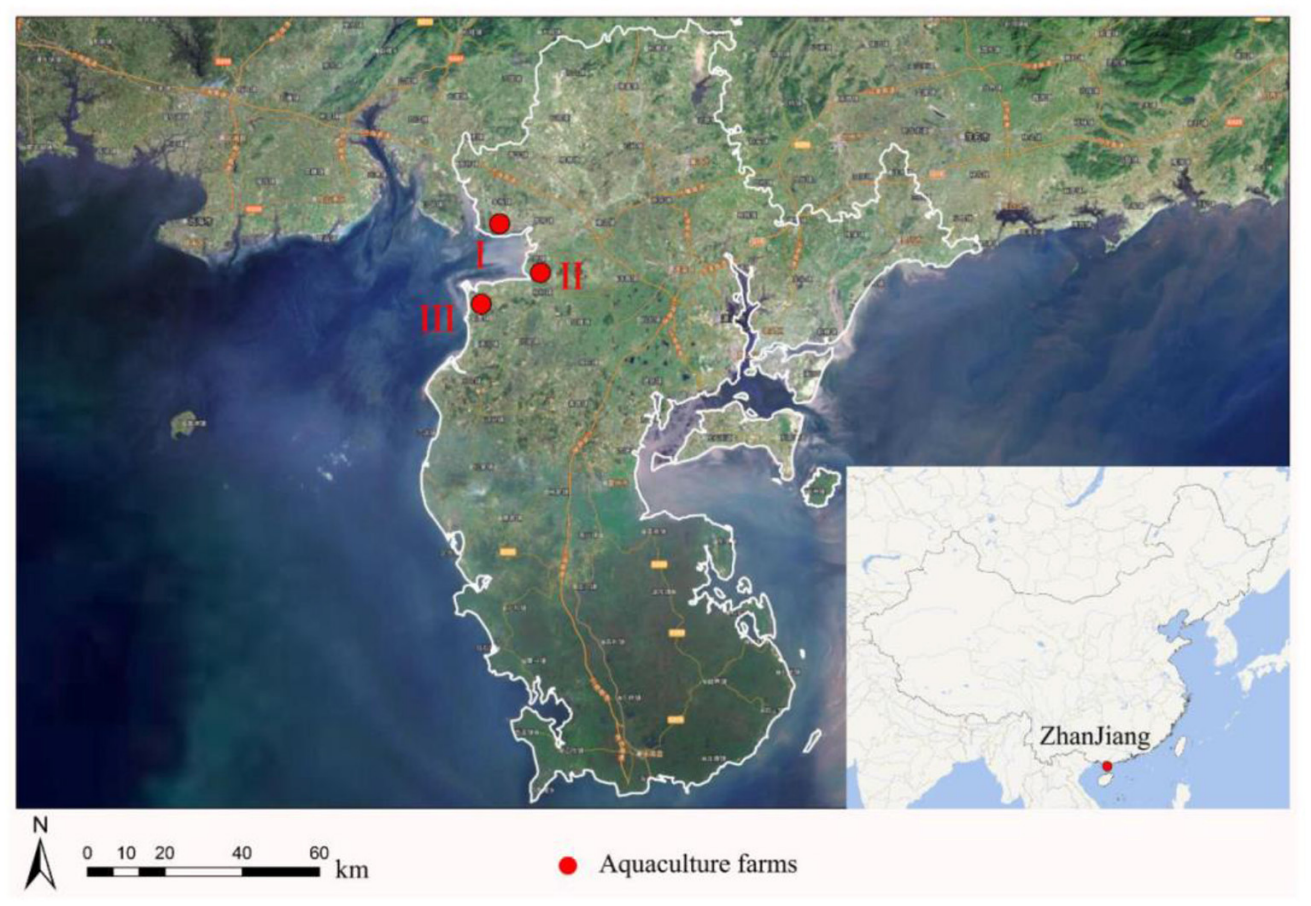
Map of the sampling locations.

The water, surrounding soil, and sediment samples were randomly collected from three aquaculture farms in Zhanjiang, China (farms I, II, and III). The sampling locations are shown in Figure 1. The water samples were collected at 1–10 cm in the water surface, the soil samples were collected at 1–3 cm on the surface of the surrounding aquaculture farms, and the sediment samples were collected at 1–5 cm from the surface of the sediment. There were 10 samples of water, soil, and sediment from each aquaculture farm, respectively, and a total of 90 samples were collected in this study.

### Reagents, Antibacterial Drugs, and Strains

MacConkey agar medium, Eosin Meilan agar medium, and ordinary nutrient broth were purchased from Beijing Luqiao Technology Co., Ltd. (Beijing, China); 5 × TBE buffers were obtained from Shenggong Bioengineering (Shanghai) Co., Ltd. (Shanghai, China); Premix EX TaqTM version 2.0 and DL1000 DNA Marker were both purchased from TaKaRa Bioengineering (Dalian) Co., Ltd. (Dalian, China); agarose (produced in Spain) was purchased from Beijing Kaioudi Biotechnology Co., Ltd. (Beijing, China); and the Goldview nucleic acid stains were from Vazyme Nanjing Biotech Co., Ltd. (Nanjing, China).

There was a total of 23 kinds of antibacterial drug sensitivity reagent tablets, including penicillin, amoxicillin, cefotaxime, ceftriaxone, aztreonam, gentamicin, amikacin, azithromycin, tetracycline, doxycycline, ciprofloxacin, ofloxacin, compound sulfamethoxazole, chloramphenicol, polymyxin B, nitrofurantoin, rifampicin, florfenicol, ampicillin, streptomycin, lomefloxacin, sulfisoxazole, and fosfomycin, which were from Chicheng Pharmaceutical Technology Co., Ltd. (Hangzhou, China). The quality control bacterial strain is *E. coli* ATCC 25922 (from the Laboratory of Basic Veterinary Medicine, Guangdong Ocean University, Zhanjiang, China).

### Isolation and Identification of *E. coli*

The collected 90 samples (water, soil, and sediment) were diluted with sterile water to a suitable quantity. Then, 100 μl was taken and spread evenly on MacConkey agar medium and incubated at 37°C for 18–24 h. A single colony with a smooth, moist, pink surface was selected and placed on eosin. Streak purification was done on blue agar medium, and the sample was cultured at 37°C for 18–24 h. A single colony with metallic luster, black or brown-green, was selected and inoculated on eosin melan agar medium for streak purification again; finally, the suspected *E. coli* was removed. The strains were inoculated into nutrient broth medium, cultivated at 37°C for 24 h, and then stored at −20°C with 30% glycerol for later analysis.

PCR method was used for the identification of isolated *E. coli* strains from the samples. The PCR primers were designed based on the specific *phoA* gene sequence of *E. coli*—upstream primer: 5′-TACAGGTGACTGCGGGCTTATC-3′, downstream primer: 5′-CTTACCGGGCAATACACTCACTA-3′–and the primers were synthesized and produced by Shenggong Bioengineering Co., Ltd. (Shanghai, China). The length of the product is 622 bp. The DNA of the isolated strains was extracted by using DNA extraction kits (Tiangen Biochemical Technology Co., Ltd., Beijing, China). The PCR reaction system is 15 μl: DNA template 1 μl, each 0.5 μl of upstream and downstream primers (25 μmol/L), and Taq 13 μl. The PCR reaction conditions were as follows: pre-denaturation at 94°C for 7 min, denaturation at 94°C for 30 s, annealing at 55°C for 30 s, and extension at 72°C for 30 s—for a total of 30 cycles, and final extension at 72°C for 5 min. The PCR products were electrophoresed on 1% agarose gel, and *E. coli* ATCC 25922 was used as standard bacteria and positive control; ddH_2_O was used as the blank negative control.

### Antimicrobial Drug Resistance Analysis of Isolated *E. coli* Strains

The drug susceptibility reagent tablet (test strips) method, which was recommended by the Clinical and Laboratory Standards Institute, was performed to detect and analyze the drug resistance of the isolated *E. coli* strains. Briefly, 90 strains of *E. coli* isolated from 90 samples were resuscitated, and 100 μl bacterial liquid was evenly spread on a common nutrient agar medium, and 23 kinds of antimicrobial drug-sensitive reagent tablets were used on the surface of the medium and then incubated at 37°C for 18–24 h. The diameter of the inhibition zone was observed and recorded, and the results were determined for drug resistance (14). There were three replicates for each antibacterial drug resistance analysis. *Escherichia coli* ATCC 25922 was used as the quality control bacteria. Finally, the resistance to each antimicrobial drug and the multi-drug resistance of the isolated *E. coli* strains from water, soil, and sediment were statistically analyzed by using Excel 2012.

### AMR Gene Detection of Isolated *E. coli* Strains

The PCR method was used to detect 29 kinds of AMR genes carried in the 90 isolated strains of *E. coli* from the water, soil, and sediment samples of the aquaculture farms. The primers are presented in Table 1 and designed according to previous studies (the reference details are described in Table 1). The PCR reaction system is 15 μl, which contained 1 μl of DNA template, 0.5 μl of upstream and downstream primers, and 13 μl of Premix EX Taq^TM^. ddH_2_O was used as blank negative control. The PCR reaction procedure was as follows: pre-denaturation at 94°C for 5 min, denaturation at 94°C for 30 s, annealing for 30 s at melting temperature (Tm, °C) (the Tm of each AMR gene is listed in Table 1), extension at 72°C for 1 min for 30 cycles, and a final extension at 72°C for 10 min. The PCR products were stored at 4°C. The PCR products were analyzed by 1% agarose gel electrophoresis, and the results were recorded.

**Table 1:** PCR primers of *Escherichia coli* antimicrobial resistance genes.

**Category**	**Genes**	**Primer sequences (5^**′**^-3^**′**^)**	**Product size (bp)**	**Tm (**°**C)**	**References**
β-Lactams	*bla_*CTX*−*M*_*	F: GGGCTGAGATGGTGACAAAGAG	876	55	(15)
		R: CGTGCGAGTTCGATTTATTCAAC			
	*bla_*TEM*_*	F: TCGCCGCATACACTATTCTCAGAATGA	445	50	(16)
		R: ACGCTCACCGGCTCCAGATTTAT			
	*bla_*CIT*_*	F: TGGCCAGAACTGACAGGCAAA	462	55	(17)
		R: TTTCTCCTGAACGTGGCTGGC			
Carbapenems	*bla_*KPC*_*	F: TCGCTAAACTCGAACAGG	785	63	(18)
		R: TTACTGCCCGTTGACGCCCAATCC			
	*bla_*DHA*_*	F: AACTTTCACAGGTGTGCTGGGT	405	55	(19)
		R: CCGTACGCATACTGGCTTTGC			
	*bla_*NDM*_*	F: GGGCAGTCGCTTCCAACGGT	475	63	(20)
		R: GTAGTGCTCAGTGTCGGCAT			
	*bla_*IMP*_*	F: CTACCGCAGCAGAGTCTTTG	587	58	
		R: AACCAGTTTTGCCTTACCAT			
Amido alcohols	*floR*	F: CTGAACACGACGCCCGCTAT	751	60	(21)
		R: GGACCGCTCCGCAAACAA			
	*cfr*	F: TGAAGTATAAAGCAGGTTGGGAGTCA	746	55	(22)
		R: ACCATATAATTGACCACAAGCAGC			
	*fexA*	F: CTCTTCTGGACAGGCTGGAA	332	57	(23)
		R: CCAGTTCCTGCTCCAAGGTA			
	*fexB*	F: ACTGGACAGGCAGGCTTAAT	320	57	
		R: CCTGCCCCAAGATACATTGC			
	*cat1*	F: AGTTGCTCAATGTACCTATAACC	547	51	(24)
		R: TTGTAATTCATTAAGCATTCTGCC			
	*OptrA*	F: CTTATGGATGGTGTGGCAGC	310	59	(25)
		R: CCATGTGGTTTGTCGGTTCA			
	*cmlA*	F: TACGACAGCGAGCACAATTC	764	54	(26)
		R: CGGTGATGGCAAGCAATACT			
Aminoglycosides	*aphA1*	F: ATGGGCTCGCGATAATGTC	634	60	(27)
		R: CTCACCGAGGCAGTTCCAT			
	*aac(3)- II*	F: GGCGACTTCACCGTTTCT	412	54	(28)
		R: GGACCGATCACCCTACGAG			
Sulfonamides	*Sul1*	F: GTGACGGTGTTCGGCATTCT	779	58	(29)
		R: CCGAGAAGGTGATTGCGCT			
	*Sul2*	F: CGGCATCGTCAACATAACCT	721	66	(30)
		R: TGTGCGGATGAAGTCAGCTC			
Tetracyclines	*tetM*	F: GAGGTCCGTCTGAACTTTGCG	915	56	(31)
		R: AGAAAGGATTTGGCGGCACT			
	*tetC*	F: CTGGGCTGCTTCCTAATGC	580	56	
		R: AGCTGTCCCTGATGGTCGT			
	*tetA*	F: GGCACCGAATGCGTATGAT	480	56	
		R: AAGCGAGCGGGTTGAGAG			
Macrolides	*ereA*	F: GCCGGTGCTCATGAACTTGAG	419	60	(32)
		R: CGACTCTATTCGATCAGAGGC			
	*ermB*	F: CGAGTGAAAAAGTACTCAACC	557	52	(33)
		R: GCCGTGTTTCATTGCTTGATG			
Quinolones	*oqxA*	F: GATCAGTCAGTGGGATAGTTT	670	51	(34)
		R: TACTCGGCGTTAACTGATTA			
	*oqxB*	F: TTCTCCCCCGGCGGGAAGTAC	512	65	(35)
		R: CTCGGCCATTTTGGCGCGTA			
	*qnrA*	F: TTCAGCAAGAGGATTTCTCA	500	55	(36)
		R: GGCAGCACTATTACTCCCAA			
	*qnrS*	F: ACGACATTCGTCAACTGCAA	417	53	(37)
		R: TAAATTGGCACCCTGTAGGC			
Colistin	*mcr1*	F: CGGTCAGTCCGTTTGTTC	309	53	(38)
		R: CTTGGTCGGTCTGTAGGG			
	*mcr2*	F: TGTTGCTTGTGCCGATTGGA	563	65	(39)
		R: AGATGGTATTGTTGGTTGCTG			

Positive fragments of the target gene were randomly picked and recovered using Poly-Gel DNA Extraction Kit (OMEGE) and cloned according to vector ligation (pMD19-T vector, TaKaRa) and competent cells (DH-5α, TaKaRa). The plasmid (Plasmid DNA Kit, OMEGE) was extracted and then sent to Shanghai Biotech, China for sequencing. The sequences were compared using BLAST (http://www.ncbi.nlm.nih.gov/blast) on the National Center for Biotechnology Information (NCBI) website. The homology between the obtained sequences and the corresponding target genes on NCBI was very high, and the matching degree was more than 98%. Ultimately, the AMR gene detection rates of the isolated *E. coli* strains from water, soil, and sediment were statistically analyzed by using Excel 2012.

### Correlation Analysis of AMR Genes and Drug Resistance in Isolated *E. coli* Strains

A correlation analysis between the results of AMR genes and drug resistance was performed, and the correlation rate of AMR genes and drug resistance was calculated. The correlation rate of AMR genes and drug resistance was calculated as to the following formula: (number of resistant strains with positive genes + number of sensitive strains with negative genes) / total number of strains × 100% (40).

## Results

### Isolation and Identification of the Isolated *E. coli* Strains

One *E. coli* strain was isolated from each sample of water, soil, and sediment, totaling to 90 strains. The colonies of the isolated strains grown on Macconkey agar medium have a smooth, moist, and pink surface, and the colonies grown on eosin melan agar medium have a metallic luster, either black or brown-green. Besides this, the PCR products of *E. coli*-specific *phoA* gene can be observed by gel electrophoresis and showed that 90 strains of bacteria have an amplified 622-bp band, and it is determined that the isolated and cultured 90 strains are *E. coli*.

### Antimicrobial Drug Resistance of Isolated *E. coli* Strains

The results of antimicrobial drug resistance of the isolated *E. coli* strains are presented in Table 2. Overall, the isolated *E. coli* strains from the three aquaculture farms all have high antimicrobial drug resistance rates to penicillin and rifampicin, and the resistance rate to rifampicin reaches 100%. The *E. coli* isolates of the water, soil, and sediment samples of the three aquaculture farms all have relatively low resistance to β-lactam aztreonam, aminoglycosides gentamicin, amikacin, streptomycin, macrolide azithromycin, doxycycline, ciprofloxacin, ofloxacin, lomefloxacin, polymyxin B, nitrofurantoin, and fosfomycin ( ≤ 50%). Specifically, the *E. coli* isolates from the water samples of aquaculture farm I have a low resistance rate to amoxicillin, ampicillin, tetracyclines, sulfonamides, and amide alcohol drugs ( ≤ 30%), but the *E. coli* isolates of the soil and sediment samples from aquaculture farms I, II, and III have high resistance rates to the above-mentioned drugs (≥60%). In addition, there is high resistance to cefotaxime and ceftriaxone for the *E. coli* isolates of the water and soil samples from aquaculture farm II (≥70%).

**Table 2:** Drug resistance rate of isolated strains of *Escherichia coli* from three aquaculture farms in Zhanjiang, China.

**Drug category**	**Drugs**	**Drug resistance rate of farm I, %**			**Drug resistance rate of farm II, %**			**Drug resistance rate of farm III, %**		
		**Water**	**Soil**	**Sediment**	**Water**	**Soil**	**Sediment**	**Water**	**Soil**	**Sediment**
β-Lactams	Penicillin	100	100	70	100	100	70	100	100	70
	Amoxicillin	30	90	70	100	100	90	100	90	90
	Ampicillin	0	100	60	100	90	90	90	80	90
	Aztreonam	0	0	0	30	0	10	10	0	10
Cephalosporins	Cefotaxime	0	0	40	90	90	40	40	10	0
	Ceftriaxone	0	10	0	90	70	40	40	0	0
Aminoglycosides	Gentamicin	0	0	0	10	30	20	20	0	0
	Amikacin	0	0	0	10	0	0	0	0	0
	Streptomycin	0	10	10	30	30	20	10	0	0
Macrolides	Azithromycin	20	20	0	30	10	10	20	0	20
Tetracyclines	Tetracycline	20	100	60	80	100	80	90	100	100
	Doxycycline	20	30	0	40	30	40	30	40	60
Quinolones	Ciprofloxacin	0	0	0	20	30	10	0	0	0
	Ofloxacin	0	0	0	10	0	10	0	0	0
	Lomefloxacin	0	10	0	20	50	20	10	0	0
Sulfonamides	Compound Sulfamethoxazole	20	80	80	80	90	100	80	90	100
	Sulfisoxazole	40	100	100	100	90	100	90	90	100
Amide alcohols	Chloramphenicol	20	100	60	90	90	100	100	100	100
	Florfenicol	20	100	80	100	90	90	100	90	100
Polymyxins	Polymyxin B	0	0	0	0	0	0	0	0	0
Nitrofurans	Nitrofurantoin	0	0	0	10	0	0	0	0	0
Rifamycins	Rifampin	100	100	100	100	100	100	100	100	100
Fosfomycins	Fosfomycin	0	0	0	0	0	0	0	0	0

The resistance of the 90 isolates of *E. coli* to 23 antibacterial drugs is shown in Table 3. The resistance rate of the 90 isolates of *E. coli* to penicillin, amoxicillin, ampicillin, tetracycline, compound sulfamethoxazole, sulfisoxazole, chloramphenicol, florfenicol, and rifampin is high (≥70%), and the resistance rate to rifampicin is 100%. The resistance rates to aztreonam, gentamicin, amikacin, streptomycin, azithromycin, ciprofloxacin, ofloxacin, lomefloxacin, polymyxin B, nitrofurantoin, and fosfomycin were low ( ≤ 30%), and the resistance rate to polymyxin B and fosfomycin was 0.

**Table 3:** Resistance of 90 isolated strains of *Escherichia coli* from three aquaculture farms in Zhanjiang, China, to 23 kinds of antimicrobial drugs.

**Antimicrobial drugs**	**Number of resistance isolates**	**Percentage, %**	**Antimicrobial drugs**	**Number of resistance isolates**	**Percentage, %**
Penicillin	81	90.00	Ciprofloxacin	6	6.67
Amoxicillin	74	82.22	Ofloxacin	2	2.22
Ampicillin	70	77.78	Lomefloxacin	11	12.22
Aztreonam	6	6.67	Compound Sulfamethoxazole	72	80.00
Cefotaxime	31	34.44			
Ceftriaxone	25	27.78	Sulfisoxazole	81	90.00
Gentamicin	8	8.89	Chloramphenicol	76	84.44
Amikacin	1	1.11	Florfenicol	77	85.56
Streptomycin	11	12.22	Polymyxin B	0	0.00
Azithromycin	13	14.44	Nitrofurantoin	1	1.11
Tetracycline	73	81.11	Rifampicin	90	100
Doxycycline	29	32.22	Fosfomycin	0	0.00

**Figure 2:**
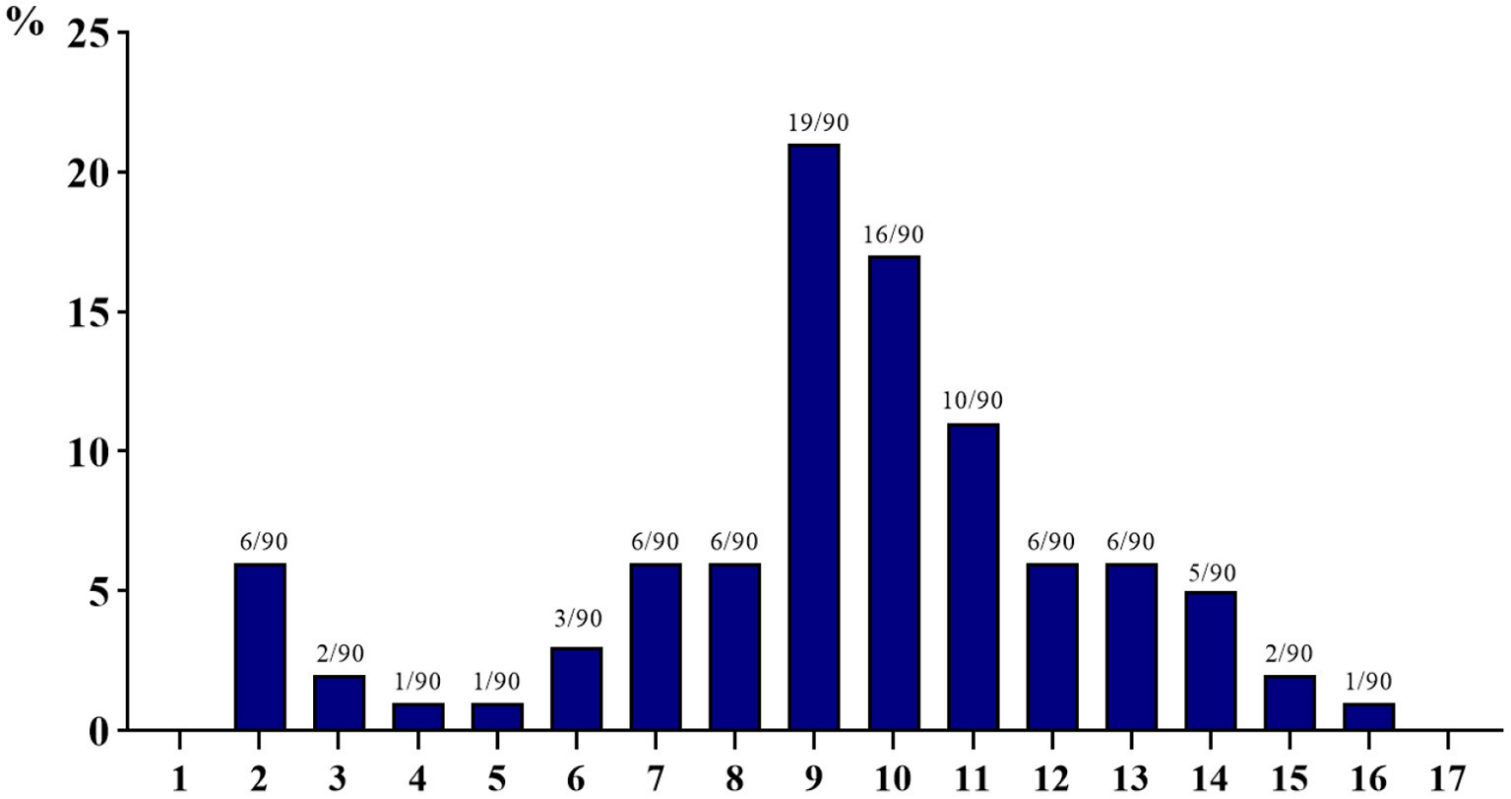
Multi-drug resistance of 90 isolated strains of *Escherichia coli* from aquaculture farms in Zhanjiang, China (the X-axis shows the number of drug resistance, while the Y-axis shows the percentage of multi-drug-resistant strains among 90 isolates).

As shown in Figure 2, all 90 isolated *E. coli* strains exhibited varying degrees of multi-drug resistance, with resistance to at least two drugs and resistance to at most 16 kinds of antibacterial drugs. Meanwhile, 60% or more isolated *E. coli* strains are resistant to more than nine drugs; especially the number of strains resistant to nine kinds of antibacterial drugs is the largest (19 strains, 21.11%), and the number of strains resistant to four, five, and 16 kinds of antibacterial drugs is the least (one strain, 1.11%).

### AMR Gene Distributions of Isolated *E. coli* Strains

The detection results of 90 strains of *E. coli* isolated from the water, soil, and sediment samples of aquaculture farms for carrying 29 kinds of AMR genes are shown in Supplementary Table 1. Regarding aquaculture farm I, the detection rate of the following AMR genes of the *E. coli* isolates from the water samples was high: *bla*_*CTX*−*M*_ (80%), *bla*_*CIT*_ (100%), *bla*_*DHA*_ (80%), *fexA* (70%), *OptrA* (90%), *aphA1* (90%) and *mcr1* (70%). The AMR genes with a high detection rate of *E. coli* isolates in soil samples include *bla*_*CIT*_ (100%), *bla*_*DHA*_ (70%), *floR* (100%), *fexB* (60%), *OptrA* (70%), *Sul2* (60%), *tetM* (90%), *oqxA* (60%), *qnrS* (100%), and *mcr1* (60%). The high detection rates of the AMR genes of *E. coli* isolates in the sediment samples are as follows: *bla*_*TEM*_(60%), *bla*_*NDM*_ (80%), *fexB* (60%), *OptrA* (90%), *cmlA* (70%), *aphA1* (70%), *Sul2* (60%), *oqxA* (90%), and *qnrS* (80%).

In aquaculture farm II, 28 kinds of AMR genes were detected, which was the largest number of detected AMR genes among the three farms. Specifically, the high detection rates of the AMR genes of *E. coli* isolates from water samples are *bla*_*CIT*_ (70%), *bla*_*DHA*_ (60%), *floR* (80%), *OptrA* (70%), *cmlA* (80%), *aphA1* (80%), *Sul2* (80%), *tetA* (70%), *qnrS* (90%), and *mcr1* (70%). The high detection rates of AMR genes of *E. coli* isolates from soil samples include *bla*_*CTX*−*M*_ (70%), *bla*_*TEM*_ (60%), *bla*_*CIT*_ (90%), *bla*_*NDM*_ (90%), *bla*_*IMP*_ (70%), *floR* (90%), *fexB* (60%), *OptrA* (80%), *cmlA* (90%), *aphA1* (60%), *Sul2* (70%), *tetC* (60%), *oqxA* (100%), *qnrS* (70%), and *mcr1* (70%). High detection rates of *E. coli* isolates in sediment samples are demonstrated for the following AMR genes: *bla*_*TEM*_ (90%), *bla*_*CIT*_ (60%), *bla*_*NDM*_ (70%), *floR* (90%), *OptrA* (70%), *Sul2* (70%), *oqxA* (80%), and *qnrS* (80%).

Regarding aquaculture farm III, high detection rates of *E. coli* isolates in water samples are observed for the following AMR genes: *bla*_*DHA*_ (70%), *floR* (80%), *fexB* (60%), *OptrA* (80%), *cmlA* (70%), *Sul2* (90%), and *qnrS* (70%). The high detection rates of AMR genes of *E. coli* isolates from soil samples include *bla*_*CTX*−*M*_ (80%), *bla*_*TEM*_ (80%), *bla*_*CIT*_ (60%), *bla*_*NDM*_ (100%), *floR* (90%), *fexA* (80%), *cmlA* (70%), *oqxA* (90%), and *qnrS* (80%). High detection rates of AMR genes of *E. coli* isolates from sediment samples are observed as follows: *bla*_*TEM*_ (70%), *bla*_*NDM*_ (100%), *floR* (100%), *OptrA* (100%), *Sul2* (90%), *oqxA* (90%), and *qnrS* (80%).

As shown in Table 4, on the whole, among the carried AMR genes in 90 strains of *E. coli* isolates, β-lactams (*bla*_*TEM*_ and *bla*_*CIT*_), amide alcohols (*floR, OptrA*, and *cmlA*), aminoglycosides (*aphA1*), sulfonamides (*Sul2*), and quinolones (*oqxA* and *qnrS*) have a high detection rate (≥50%). The detection rate of AMR genes of carbapenems (*bla*_*KPC*_ and *bla*_*IMP*_), amide alcohols (*cfr* and *cat1*), aminoglycosides (*aac(3)-II*), sulfonamides (*Sul1*), tetracyclines (*tetM* and *tetA*), macrolides (*ereA* and *ermB*), quinolones (*oqxA* and *qnrB*), and colistins (*mcr2*) is relatively low ( ≤ 30%), and the detection rate of *mcr2* is 0.

**Table 4:** Detection of antimicrobial resistance genes in a total of 90 isolated strains of *Escherichia coli* from aquaculture farms in Zhanjiang, China.

**Antimicrobial resistance genes**	**Number of resistance isolates**	**Detection rate %**	**Antimicrobial resistance genes**	**Number of resistance isolates**	**Detection rate %**
*bla_*CTX*−*M*_*	40	44.44	*aac(3)- II*	10	11.11
*bla_*TEM*_*	50	55.55	*Sul1*	5	5.56
*bla_*CIT*_*	61	67.77	*Sul2*	59	65.56
*bla_*KPC*_*	4	4.44	*tetM*	14	15.56
*bla_*DHA*_*	41	45.55	*tetC*	28	31.11
*bla_*NDM*_*	54	60.00	*tetA*	20	22.22
*bla_*IMP*_*	22	24.44	*ereA*	1	1.11
*floR*	74	82.22	*ermB*	17	18.89
*Cfr*	10	11.11	*oqxA*	53	58.89
*fexA*	27	30.00	*oqxB*	8	8.89
*fexB*	32	35.56	*qnrA*	6	6.67
*cat1*	19	21.11	*qnrS*	66	73.33
*OptrA*	70	77.78	*mcr1*	41	45.56
*cmlA*	55	61.11	*mcr2*	0	0.00
*aphA1*	51	56.67			

**Figure 3:**
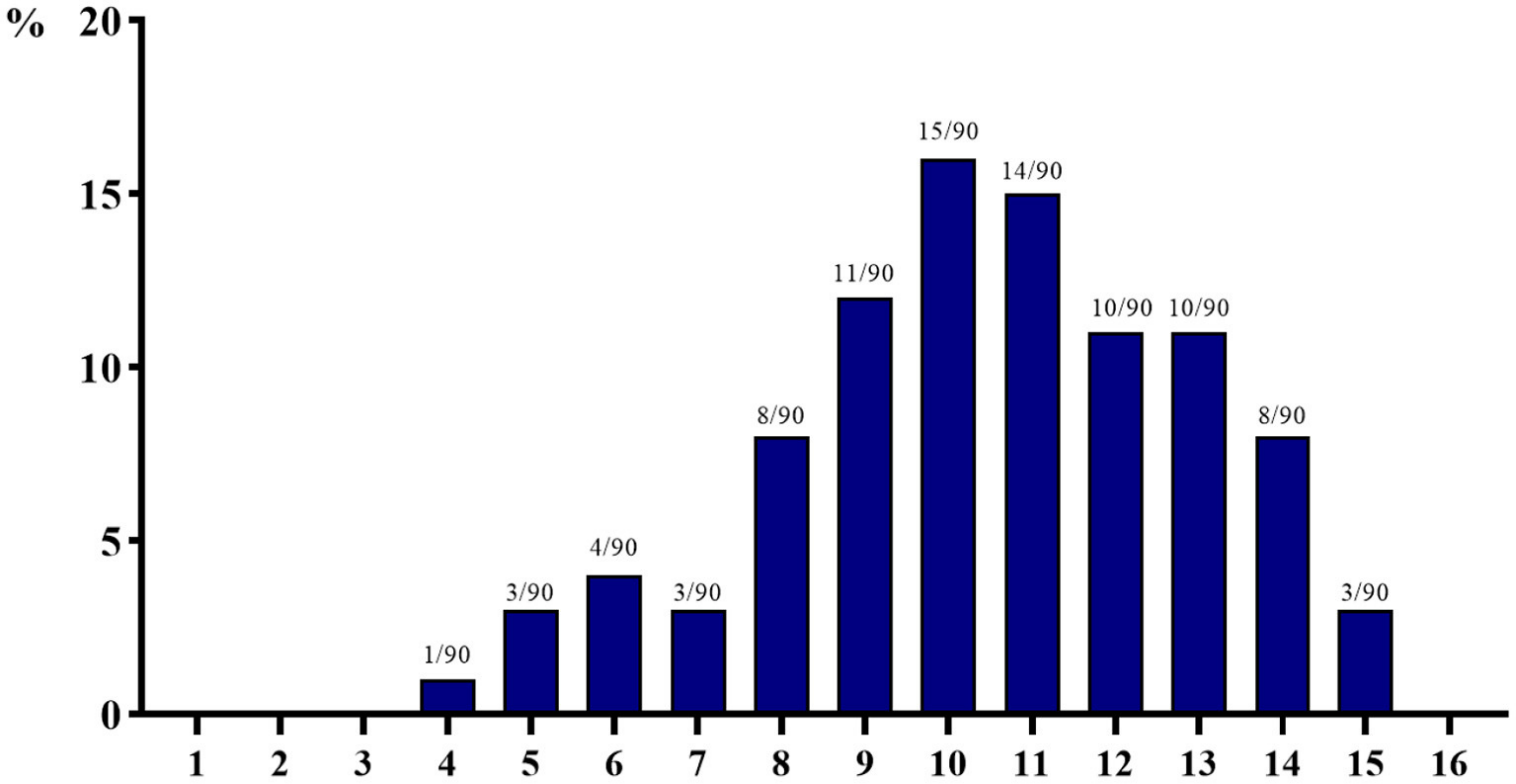
The number of 29 antimicrobial resistance genes detected in 90 isolated strains of *Escherichia coli* from aquaculture farms in Zhanjiang, China (the X-axis indicates the number of resistance genes, while the Y-axis indicates the percentage of bacteria carrying multi-drug resistance genes among 90 isolates).

The number and detection rate of 29 AMR genes in 90 isolated *E. coli* strains are shown in Figure 3. AMR genes of at least four species were detected, and a maximum of 15 AMR genes were detected in each strain of isolated *E. coli*. More than 60% of the isolated *E. coli* strains carried more than 10 kinds of AMR genes, of which the number of isolated *E. coli* strains carrying 10 kinds of AMR genes is the largest (15 strains, 15.56%), and the number of isolated *E. coli* strains carrying four kinds of AMR genes is the least (one strain, 1.11%).

### Correlation Analysis of AMR Genes and Drug Resistance

As described in Table 5, the following AMR genes have a high correlation rate with the drug resistance of the isolated *E. coli* strains: *bla*_*TEM*_ (55.56%), *bla*_*CIT*_ (67.78%), *floR* (85.56%), *OptrA* (71.11%), *cmlA* (64.44%), *aac(3)-II* (81.11%), *Sul2* (70 %), *ereA* (84.44%), *ermB* (73.33%), *oqxB* (85.56%), *qnrA* (83.33%), *mcr1* (56.67%), and *mcr2* (95.56%). The correlation rate of other AMR genes with drug resistance is relatively low (<50%).

**Table 5:** Correlation analysis between antimicrobial resistance genes and drug resistance of *Escherichia coli* isolates from aquaculture farms in Zhanjiang, China.

**Category**	**Test of antimicrobial resistance genes**		**Test of drug resistance**		**Correlation rate, %**
	**Genes**	**Negative (N)/positive (P)**	**Number of detections**	**Sensitive strain**	
β-lactams	*bla_*TEM*_*	P	50	0	55.56
		N	40	0	
	*bla_*CIT*_*	P	61	0	67.78
		N	29	0	
Amide alcohols	*floR*	P	70	4	85.56
		N	9	7	
	*OptrA*	P	62	9	71.11
		N	17	2	
	*cmlA*	P	51	4	64.44
		N	28	7	
Aminoglycosides	*aac(3)- II*	P	7	3	81.11
		N	14	66	
Sulfonamides	*Sul2*	P	56	3	70.00
		N	24	7	
Macrolides	*ereA*	P	0	1	84.44
		N	13	76	
	*ermB*	P	3	14	73.33
		N	10	63	
Quinolones	*oqxB*	P	3	5	85.56
		N	8	74	
	*qnrA*	P	1	5	83.33
		N	10	74	
Colistin	*mcr1*	P	3	38	56.67
		N	1	48	
	*mcr2*	P	0	0	95.56
		N	4	86	

*Only the data with a correlation rate of >50% are presented*.

## Discussion

China is a huge market for animal product consumption. With the increase in consumer demand for meat products other than livestock products, the scale of aquaculture has significantly increased in recent years, and Zhanjiang is an important place for aquatic product production in China (2). However, in order to improve the economic benefits of aquaculture, antimicrobial drugs (including preventive and therapeutic drugs) are widely used in the aquaculture industry to prevent and treat bacterial diseases (1). The overuse and/or abuse of antimicrobial drugs in animal production leads to the generation of AMR genes and drug-resistant bacteria, thus threatening to complicate the treatment of bacterial infections in humans (8, 9). Due to the fact that *E. coli* is a common pathogen in both humans and animals, the AMR issue of *E. coli* has been a prominent concern in public health (10, 11). Accordingly, the application of antibiotics for growth promotion in farm animals was banned entirely in the European Union since 2000 and in South Korea and Japan after 2011 (41). Recently, China also completely banned the preventive antibiotics for promoting the growth performance of animals to mitigate the deleterious consequences of AMR to public health and encouraged research on alternatives to preventive antibiotics (42–45). Nevertheless, in large-scale aquaculture, the extensive use of therapeutic antibacterial drugs induced the AMR issues and was still a major concern. In this context, the present study investigated the distribution of AMR genes and analyzed the drug resistance of *E. coli* in aquaculture farms of Zhanjiang and their surrounding environments, and the findings have positive implications for public health.

Through the drug resistance analysis of the present study, it was found that the *E. coli* strains isolated from three aquaculture farms in Zhanjiang, China, have different levels of resistance to various antimicrobial drugs. The *E. coli* isolates from aquaculture environments are highly resistant to β-lactams (except aztreonam), tetracycline, sulfonamides, amide alcohols, and rifamycin but have a relatively low resistance rate to aztreonam, aminoglycosides, macrolides, doxycycline, quinolones, polymyxins, nitrofurans, and fosfomycins. It is worth noting that the 90 isolated *E. coli* strains in this study were resistant to at least two drugs and at most 16 drugs, indicating that the multi-drug resistance of *E. coli* was a serious concern in the aquaculture farms of Zhanjiang, China. Similarly, Saharan et al. (46) reported that the majority of the *E. coli* isolates from a fish farm in India were found to be resistant to more than three antibiotics (>89%), suggesting that there was multi-drug resistance for *E. coli* in the fish farm. According to the study of Ng et al. (47), it was found that 33 multi-drug-resistant *E. coli* were isolated from surface waters in aquaculture sites of Singapore. Lihan et al. (48) investigated the antibiotic-resistant *E. coli* from Sarawak rivers and aquaculture farms in northwest of Borneo. They found that resistance to piperacillin (100%) was observed in all *E. coli* isolates, resistance to amoxicillin (100%) and ampicillin (100%) was observed in *E. coli* isolated from aquaculture farms, and resistance to streptomycin (93%) was observed in *E. coli* isolated from rivers. The rivers and aquaculture farms showed similar partial drug resistance, indicating that the drug-resistant *E. coli* produced by aquaculture can be transferred to surrounding waters. On the other hand, compared to these previous reports, different aquaculture farms showed different degrees of resistance to various antibacterial drugs. This may be due to a variety of feed formulas and various medication regimes of the aquaculture farms in different regions.

Nowadays, the AMR genes are considered as new environmental pollutants; the AMR genes not only spread to surrounding environments (such as water) and the food chain but also spread to other bacteria horizontally through genetic elements, leading to antibiotic-resistant bacteria and causing difficulties in therapy. Therefore, the AMR in bacteria associated with food and water has been a global concern (7, 8). Previously, it has been reported that multiple AMR genes (mainly *sul1, sul2*, and *tetM*) were detected in *E. coli* isolates from surface water samples of aquaculture farms in America (49). Hassan et al. (50) also found that around five strains of *E. coli* isolated from a rainbow trout farm carried the *mcr-1* gene. In this study, the 90 strains of *E. coli* isolates from three aquaculture farms in Zhanjiang, China, mainly carried the AMR genes of β-lactams (*bla*_*TEM*_ and *bla*_*CIT*_), carbapenems (*bla*_*NDM*_), amide alcohols (*floR, OptrA*, and *cmlA*), aminoglycosides (*aphA1*), sulfonamides (*sul2*), and quinolones (*oqxA* and *qnrS*), among which *floR* has the highest carrying rate (82.2%). It was consistently found that the *floR* gene detection rate in the isolates from aquaculture farms in coastal areas of Jiangsu, China, was 76.67% (51). By contrast, Ng *et al*. (47) demonstrated that there was high relative abundance of *sul1, qnrA*, and *intI1* genes in the sediments of aquaculture farms in Singapore. Changkaew et al. (52) suggested that the *tet(A)* gene was the most common AMR gene (69.1%) in the 55 *E. coli* isolates from shrimp farms and their surrounding environment in Thailand. Ng et al. (53) isolated 19 strains of *E. coli* from 10 aquaculture farms and their environment in Malaysia, and they found that the AMR gene of *Cat I* has the highest detection rate (100%, 19 of 19). On the basis of these reports, we can clearly realize that there were various AMR genes carried by *E. coli* in aquaculture farms of different regions. This may be because of the different epidemic diseases of aquatic animals and the variety of medication regimens that were executed in these regions, thus resulting in the generation of inconsistent AMR genes. Therefore, the contamination of AMR genes in the aquaculture industry of different regions requires investigation case by case. Most notably, the *floR* gene had the highest detection rate in this experiment, which has been confirmed to be the main AMR gene that mediates drug resistance in *E. coli* (54). Thus, the aquaculture farms in Zhanjiang area should avoid the overuse of amide alcohol drugs. Besides this, the 90 strains of *E. coli* isolates in this study have a low carrying rate of tetracycline (*tetM, tetC*, and *tetA*), macrolide (*ereA* and *ermB*), and colistin (*mcr1* and *mcr2*), and none of the AMR genes was detected in the 90 *E. coli* isolates at the same time. Bacteria have different resistance mechanisms to different antimicrobial drugs, for instance, tetracycline-resistant bacteria usually produce a protein that interacts with ribosomes; thereby, protein synthesis is not affected by the antimicrobial drugs, which is called ribosome protection (36). *Escherichia coli* can produce macrolide phosphotransferase, which destroys the lactone ring of macrolide antibiotics, resulting in a high degree of resistance to macrolide drugs (27). Moreover, the detection rate of *mcr1* was not high (45.56%), and the *mcr2* gene was not detected among the 90 *E. coli* isolates from aquaculture farms. The possible reasons are as follows: (1) *mcr1* is a relatively universal AMR gene in animals, but the *mcr2* gene was carried by a rare plasmid (IncX4 type), and (2) the colistin drugs are one of the “last resorts” for the treatment of multi-drug resistant gram-negative bacterial infections; therefore, the aquaculture farms use this type of drug less.

In this study, the AMR genes of β-lactams (*bla*_*TEM*_ and *bla*_*CIT*_), amide alcohols (*floR, OptrA*, and *cmlA*), aminoglycosides [*aac(3)-II*], sulfonamides (*sul2*), macrolides (*ereA* and *ermB*), quinolones (*oqxB* and *qnrA*), and colistin (*mcr1* and *mcr2*) are highly correlated with their drug resistance (>55%), while the other AMR genes are relatively less related to their drug resistance. *E. coli* has a strong ability to accumulate AMR genes. Previous studies have shown that *E. coli* from animals often carry multiple AMR genes, but not every AMR gene exhibits corresponding resistance (34, 55). There are many reasons for the development of drug resistance in bacteria, which are related to the characteristics of the bacteria, the spread of AMR genes, and the use of drugs (1, 3). Among them, although the most important factor of the AMR gene, the expression of AMR genes was associated with the complex regulation by multiple substances (5–7). Therefore, the AMR genes are detected in some bacteria, but the related drug resistance phenotypes may not be shown because the AMR genes are not expressed or are expressed in low abundance (7, 55). Exploring the intrinsic relationship between AMR genes and drug resistance and reducing the spread of AMR genes from the inner roots to curb the trend of drug resistance still require further in-depth research.

## Conclusion

Collectively, the *E. coli* isolated from the aquaculture farms and their environment in Zhanjiang, China, showed multi-drug resistance and carried a large number of AMR genes. Among them, the *floR* gene had the highest detection rate. Meanwhile, there was a high correlation between some of the AMR genes and the drug resistance phenotypes. Therefore, in order to reduce the spread of AMR genes and the production of drug-resistant bacteria, the present study suggests for aquaculture practitioners in Zhanjiang area to decrease the use of amide alcohol drugs and regulate the use of various antibacterial drugs. The current findings persuade the prudent use of antimicrobial agents in aquatic animal farming and also provide basic information for better understanding of AMR in aquaculture farms in Zhanjiang, China, thus being beneficial to public health.

## Data Availability Statement

The original contributions presented in the study are included in the article/Supplementary Material, further inquiries can be directed to the corresponding authors.

## Author Contributions

W-CL, YM, and BB: conceptualization, writing—review, and editing. C-YL and J-JP: methodology and data curation. C-YL, J-JP, and S-RT: analysis. W-CL, C-YL, and BB: writing—original draft preparation. YM: supervision, project administration, and funding acquisition. All authors contributed to the article and approved the submitted version.

## Funding

This research was supported by the 2019 Guangdong University Features Innovation Project by the Department of Education in Guangdong Province, China (2019KTSCX057), the Project of Innovation and Strengthening of Guangdong Ocean University (Q18290), and Guangdong Provincial Department of Education 2021 Special Project for Key Fields of Ordinary Colleges and Universities (2021ZDZX4003).

## Conflict of Interest

The authors declare that the research was conducted in the absence of any commercial or financial relationships that could be construed as a potential conflict of interest.

## Publisher's Note

All claims expressed in this article are solely those of the authors and do not necessarily represent those of their affiliated organizations, or those of the publisher, the editors and the reviewers. Any product that may be evaluated in this article, or claim that may be made by its manufacturer, is not guaranteed or endorsed by the publisher.
