# Supplementary material for: Antimicrobial Resistance of Escherichia coli From Aquaculture Farms and Their Environment in Zhanjiang, China
Source: Front Vet Sci. 2021 Dec 24;8:806653. doi: 10.3389/fvets.2021.806653 (PMC8740034; doi:10.3389/fvets.2021.806653)
Supplement: Supplementary file 1 [file Table_1.docx]

**SUPPLEMENTAL TABLE 1** Detection rate of antimicrobial resistance genes in isolated strains of *Escherichia coli* from different samples three aquaculture farms in Zhanjiang, China

| Category | Genes | Detection rate of farm I, % | | | Detection rate of farm II % | | | Detection rate of farm II, % | | |
| --- | --- | --- | --- | --- | --- | --- | --- | --- | --- | --- |
|  |  | water | soil | sediment | water | soil | sediment | water | soil | sediment |
| β-lactams | *bla_CTX-M_* | 80 | 0 | 30 | 20 | 70 | 40 | 30 | 80 | 50 |
|  | *bla_TEM_* | 20 | 50 | 60 | 30 | 60 | 90 | 40 | 80 | 70 |
|  | *bla_CIT_* | 100 | 100 | 30 | 70 | 90 | 60 | 50 | 60 | 50 |
| Carbapenems | *bla_KPC_* | 0 | 0 | 0 | 0 | 0 | 30 | 0 | 0 | 10 |
|  | *bla_DHA_* | 80 | 70 | 0 | 60 | 0 | 50 | 70 | 30 | 50 |
|  | *bla_NDM_* | 10 | 50 | 80 | 10 | 90 | 70 | 30 | 100 | 100 |
|  | *bla_IMP_* | 30 | 30 | 20 | 0 | 70 | 10 | 10 | 40 | 10 |
| Amide alcohols | *floR* | 60 | 100 | 50 | 80 | 90 | 90 | 80 | 90 | 100 |
|  | *cfr* | 0 | 0 | 0 | 30 | 0 | 0 | 10 | 30 | 30 |
|  | *fexA* | 70 | 10 | 40 | 30 | 10 | 10 | 20 | 80 | 0 |
|  | *fexB* | 0 | 60 | 60 | 0 | 60 | 10 | 60 | 50 | 20 |
|  | *cat1* | 40 | 0 | 30 | 20 | 40 | 10 | 20 | 30 | 0 |
|  | *OptrA* | 90 | 70 | 90 | 70 | 80 | 70 | 80 | 50 | 100 |
|  | *cmlA* | 30 | 50 | 70 | 80 | 90 | 40 | 70 | 70 | 50 |
| Aminoglycosides | *aphA1* | 90 | 50 | 70 | 80 | 60 | 30 | 50 | 50 | 30 |
|  | *aac(3)- Ⅱ* | 0 | 0 | 0 | 10 | 20 | 30 | 40 | 0 | 0 |
| Sulfonamides | *Sul1* | 20 | 0 | 0 | 20 | 0 | 0 | 10 | 0 | 0 |
|  | *Sul2* | 30 | 60 | 60 | 80 | 70 | 70 | 90 | 40 | 90 |
| Tetracyclines | *tetM* | 0 | 90 | 0 | 0 | 40 | 0 | 0 | 10 | 0 |
|  | *tetC* | 20 | 20 | 40 | 50 | 60 | 30 | 30 | 30 | 0 |
|  | *tetA* | 10 | 0 | 50 | 70 | 30 | 30 | 0 | 0 | 10 |
| Macrolides | *ereA* | 0 | 0 | 0 | 10 | 0 | 0 | 0 | 0 | 0 |
|  | *ermB* | 0 | 10 | 20 | 40 | 30 | 10 | 50 | 10 | 0 |
| Quinolones | *oqxA* | 0 | 60 | 90 | 20 | 100 | 80 | 0 | 90 | 90 |
|  | *oqxB* | 10 | 0 | 0 | 10 | 20 | 10 | 20 | 10 | 0 |
|  | *qnrA* | 0 | 30 | 0 | 0 | 10 | 0 | 10 | 10 | 0 |
|  | *qnrS* | 10 | 100 | 80 | 90 | 70 | 80 | 70 | 80 | 80 |
| Colistin | *mcr1* | 70 | 60 | 10 | 70 | 70 | 50 | 30 | 40 | 10 |
|  | *mcr2* | 0 | 0 | 0 | 0 | 0 | 0 | 0 | 0 | 0 |
